# Effect of 3-Mercaptopropyltriethoxysilane Modified Illite on the Reinforcement of SBR

**DOI:** 10.3390/ma15103459

**Published:** 2022-05-11

**Authors:** Zhepeng Wang, Hao Zhang, Qiang Liu, Shaojuan Wang, Shouke Yan

**Affiliations:** 1 Key Laboratory of Rubber-Plastics, Ministry of Education, Qingdao University of Science & Technology, Qingdao 266042, China; wzhepeng@163.com (Z.W.); liuqiang@qust.edu.cn (Q.L.); wangshaojuan1982@163.com (S.W.); 2 State Key Laboratory of Chemical Resource Engineering, Beijing University of Chemical Technology, Beijing 100029, China

**Keywords:** illite, SBR, KH580, particle size, filler–rubber interaction

## Abstract

To achieve the sustainable development of the rubber industry, the substitute of carbon black, the most widely used but non-renewable filler produced from petroleum, has been considered one of the most effective ways. The naturally occurring illite with higher aspect ratio can be easily obtained in large amounts at lower cost and with lower energy consumption. Therefore, the expansion of its application in advanced materials is of great significance. To explore their potential use as an additive for reinforcing rubber, styrene butadiene rubber (SBR) composites with illites of different size with and without 3-mercaptopropyltriethoxysilane (KH580) modification were studied. It was found that the modification of illite by KH580 increases the K-illite/SBR interaction, and thus improves the dispersion of K-illite in the SBR matrix. The better dispersion of smaller size K-illite with stronger interfacial interaction improves the mechanical properties of SBR remarkably, by an increment of about nine times the tensile strength and more than ten times the modulus. These results demonstrate, except for the evident effect of particle size, the great importance of filler–rubber interaction on the performance of SBR composites. This may be of great significance for the potential wide use of the abundant naturally occurring illite as substitute filler for the rubber industry.

## 1. Introduction

Illite, a typical clay mineral belonging to the family of 2:1 layered silicates with a crystal structure of two silicon−oxygen tetrahedrons fused to an edge-shared aluminum−oxygen octahedron, has huge reserves and low production cost [1,2,3]. Owing to its moderate cation exchange capacity and large specific surface area, it is now most widely used in the fields of adsorption of dyes, perchlorate, and antibiotics in aqueous environment [4,5,6], remediation of soil [7], or removal of hazardous radionuclides or toxic heavy metal pollutants [8,9]. Illite belongs to the mica group [10]. It has yet a higher aspect ratio, a vital factor influencing the application of clay in polymers [11], than other clays, such as the most commonly used smectite-type montmorillonite [12]. This may be able to endow the polymer composites with desired performance. Therefore, attempts for its application in thermal plastic polymer composites have been made [13,14,15,16,17,18]. It has been proven that the addition of illite or modified illite can improve the thermal stability of high-density polyethylene and isotactic polypropylene (iPP), attributed to the excellent chemical stability of illite [13,14,15]. Besides, fluorinated illite filled iPP composites exhibit a 50% enhancement in flame retardancy compared to the neat iPP [15]. It has also been found that incorporation of only a small amount illite, modified by aluminate coupling agent, can toughen the rigid polyvinyl chloride significantly [16]. However, to the best of our knowledge, very few studies have been performed on the use of illite as an additive for rubber matrix, which is a typical and important polymer material.

Rubbers with high elasticity play an irreplaceable role in various fields. However, the application of rubbers must be reinforced with filler to enhance their mechanical strength. Carbon black and silica are the two most widely used reinforcing fillers for rubber [19]. However, their energy requirements in the manufacturing process are high [19]. In addition, carbon black is a non-renewable resource derived from petroleum, while the production of silica requires the use of harsh chemicals and high temperatures [20]. To achieve the goal of sustainable development in rubber industry, many new fillers have been employed. Among them, the abundant clays such as kaolinite [21,22], montmorillonite [23,24], hydrotalcite [25,26], vermiculite [27,28], sepiolite [29,30], and illite [31,32,33,34,35,36,37,38,39] have drawn great attention because of their low production price and carbon footprints. The reinforcement effect of these clays on rubber matrix is, however, not satisfactory. Therefore, a mass of efforts have been devoted to the promotion of reinforcement effect of clays, especially the silane modification of clays. For example, Yang et al. [22] constructed chemical bonds between kaolinite and styrene butadiene rubber (SBR) by the incorporation of 3-[2-(2-aminoethyl-amino)ethylamino]propyl-trimethoxy-silane (KH892). The dispersion state of kaolinite in SBR was significantly improved and the mechanical performances of composite were enhanced. Zhang et al. [24] found that the mechanical properties of organophilic montmorillonite (OMMT) filled nitrile butadiene rubber (NBR) nanocomposites could be improved by the coupling agents of γ-(mercaptopropyl) triethoxy silane and bis-[(γ-triethoxy silane)proply] tetrasulfur, but reduced by γ-(aminopropyl)triethoxysilane. Zhang et al. [25] grafted γ-aminopropyltriethoxysilane (APS) onto dodecyl sulphate-modified hydrotalcite (Mg-Al LDH) and added it to polyurethane (PU). The enhanced interfacial interaction between APS-DS-LDH and PU rubber resulted in sufficient reinforcement of the composite properties. Chen et al. [28] improved the reinforcing effect of vermiculite on SBR by the use of bis [3-(triethoxysilyl)propyl] tetrasulfide. As for the illite, silane coupling agent, titanate coupling agent, triethanolamine/fatty acid and metal acetates have been used for its modification [31,32,33,34,35,36]. The desired mechanical properties have been obtained when the modified illite was incorporated into the rubber or rubber blend, and the composites have already been used in products such as the hose, the inner tube, and the cover of motortrucks [37,38]. For rubber composites filled with other fillers, reduced filler size and filler network, improved filler dispersion, enhanced filler–rubber interaction, and composite crosslinking density have been proven to contribute to the promotion of their mechanical properties [40,41,42,43]. However, those researches about the illite filled rubber composites mainly focus on the mechanical properties and their application in industry, whereas these factors related to the reinforcement of illite on rubber are far less investigated. On the other hand, these studies using illite as a filler are mostly based on the natural rubber (NR) or its blend with other rubbers. NR is a self-reinforcing rubber due to the strain-induced crystallization, on which the reinforcing effect of the same illite is less obvious than that of non-self-reinforcing rubber such as SBR, which was chosen as the rubber matrix in this work.

Illite is a naturally-occurring clay mineral. The distinction between the different types of illite mainly lies in their locality. The illite used is produced from Chengde, Heibei province, in which the known reserves of illite (more than 1 billion tons) are the largest in China. The huge reserves endow this illite with a cheaper price, which can greatly reduce the production cost of rubber products when it is used as a filler. In a previous work [44], the possibility of illite and hexadecyl trimethyl ammonium bromide (CTAB) modified counterpart as substitute filler for natural rubber (NR) has been explored. It is found that pristine illite does not reinforce NR remarkably, which is related to the weak interfacial interaction between illite and NR. The modification of illite by CTAB enhances the interfacial interaction of it with NR and thus improves the properties of NR. Also, the capability of illite, either modified by 3-mercaptopropyltriethoxysilane (KH580) or bis[3-(triethoxysilyl)propyl] tetrasulfide (Si69), as a substitute filler for reinforcing of styrene butadiene rubber (SBR), has been investigated and the KH580 was proved to have a better modification effect on illite than Si69 [45], which is the most widely used silane coupling agent in rubber industry. Therefore, the effect of illite particle size on the interface as well as the properties of KH580-modified illite/SBR composites was further illustrated in this work. It is found that the modification of illite by KH580 increases the K-illite/SBR interaction, and thus improves the dispersion of K-illite in the SBR matrix. Combined with the smaller size, the better dispersion of K-illite with stronger interfacial interaction improves the mechanical properties of SBR significantly. This work may pave the way for expanding the use of illite in the rubber industry.

## 2. Materials and Methods

### 2.1. Materials

SBR (a copolymer elastomer synthesized from butadiene and styrene monomers, the content of styrene is 23.7 wt%) latex (a stable colloidal dispersion of butadiene styrene rubber particles in an aqueous medium, the mass concentration of dry SBR in this SBR latex, namely solid content, is 20 wt%) was supplied by Four Bung Industrial Co., Ltd., Hangzhou, China. Three kinds of illites with different sizes denoted as illite-x with x = 1, 2, 3 were kindly supplied by Chengde Renhe Mining Co., Ltd., Chengde, China. Another one named as illite-4 was obtained by ultrasonic treatment of the illite-3; 3-mercaptopropyltriethoxysilane (KH580) was produced by Huaian Heyuan Chemical Co., Ltd., Huaian, China. Calcium chloride (CaCl_2_, content: ≥96.0%) and toluene (content: ≥99.5%) were purchased from Sinopharm Chemical Reagent Co., Ltd. (Shanghai, China) Zinc oxide (ZnO), stearic acid (SA), N-tert-butylbenzothiazole-2-sulphenamide (NS), and sulfur (S) were all purchased from Jiatuo Chemical Technology Co., Ltd., Shijiazhuang, China. All of the materials were used as received without any pre-history.

### 2.2. Modification of Illite

The modification of illite with KH580 was performed by adding 1 wt% KH580 into a 10 wt% illite water suspension and stirring for 12 h at 80 °C. The obtained slurry denoted as K-illite was then used to prepare SBR composites. This procedure has also been employed to the silane grafting of silica [46], the silane modification of Bent [47] and so on.

### 2.3. Preparation of Illite-SBR and K-Illite-SBR Masterbatches

Masterbatches of illite-SBR and K-illite-SBR were prepared through the latex blending method, which could promote the dispersion of filler in SBR matrix and ensure a high coupling efficiency of KH580 [46]. To this end, 10 wt% water slurry of illite or K-illite was added into SBR latex under constant mixing at 25 °C. The mass ratio of illite or K-illite to SBR was 2 to 5. The mass of SBR latex used was calculated according to its solid content. After 30 min of magnetic stirring at 500 rpm, the mixture was coagulated using CaCl_2_ solution (5 wt%) and then washed with deionized water repeatedly and dehydrated at 60 °C for 48 h. The resultant masterbatches were denoted as illite-x-SBR or K-illite-x-SBR with the x = 1, 2, 3, 4, representing the used illite.

### 2.4. Preparation of SBR Composites

A basic formulation used for all SBR composites (also the unfilled SBR as a reference material) is shown in Table 1. The preparation of SBR compounds was realized on a two-roll mill (BL-6175-AL, Donguan Bolon Precision Testing Machines Co., Ltd., China. Roll diameter: 150 mm. Roll width: 320 mm. Roll gap: adjustable from 0.05 to 8 mm. Friction ratio: 1:1.4). The related SBR masterbatch was first masticated for 10 min. Then ZnO, SA, and NS were added to the SBR masterbatch, followed by the addition of sulfur. The compounds were sheeted out after the masterbatch and other rubber auxiliaries were well mixed. The resultant SBR compounds were parked overnight and then compression-molded at 160 °C to produce SBR vulcanizates. The optimum curing time (t_90_) determined by a moving die rheometer was taken as the curing time.

### 2.5. Characterization

#### 2.5.1. Particle Size Distribution and Specific Surface Area

Illite particle size distribution was measured by laser light scatterometer (DAWN HELEOS, Wyatt Technology, Goleta, CA, USA). The specific surface area (S_BET_) of illite was determined by the N_2_ adsorption–desorption machine (ASAP 2420, Micromeritics, Norcross, GA, USA) and calculated through Brunauer–Emmett–Teller (BET) method. According to the classification of isotherms, the illite adsorption isotherm of N_2_ conforms to Type I (Langmuir) adsorption behavior.

#### 2.5.2. FTIR

FTIR was carried out for the characterization of raw illite and K-illite on a Fourier Transform Infrared spectrometer (VERTEX 70, Bruker, Germany). The specimens were prepared through the KBr pressed-disk technique (1 mg of dry illite or K-illite and 200 mg of KBr); 32 scans were acquired for each spectrum at a 4 cm^−1^ resolution in the range of 4000 to 400 cm^−1^.

#### 2.5.3. Mechanical Properties

Tensile and tear tests were carried out on a universal material testing machine (INSTRON 5943, INSTRON, UK) using an extension rate of 500 mm/min according to ISO 37-2005 and ISO 34-1-2015 standards, respectively. Tensile tests were done on dumb-bell pieces (Type 2) with a size of 75 mm × 4 mm × 2 mm, while tear tests were conducted on angle test pieces with a size of 100 mm × 20 mm × 2 mm. The pieces were cut from the vulcanizate sheet of 2 mm. Five replicates of each piece were tested for tensile and tear measurements and the mean value with the statistical error was presented.

#### 2.5.4. Crosslinking Density

The crosslinking density ($V_{e}$) was measured by equilibrium swelling method [48] with the solvent of toluene and calculated according to Flory–Rehner equation as follows:

(1) $$V_{e}=\frac{-1}{v}\left[\frac{\ln\left(1-V_{r}\right)+V_{r}+\chi V_{r}^{2}}{V_{r}^{1/3}-V_{r}/2}\right]$$

where v is the molar volume of the solvent (the value for toluene is 106.5 mL/mol). $V_{r}$ is the volume fraction of SBR in the swollen rubber vulcanizate. χ is the Flory–Huggins parameter of polymer–solvent interaction (0.446 for SBR–toluene [49]). The sample of about 100 mg was cut from the vulcanizate sheet. Three samples of each piece were used for the crosslinking density measurements and the mean value with the statistical error was presented.

#### 2.5.5. DSC

The specific heat capacity curves showing the glass transition process were acquired through the differential scanning calorimeter test (DSC 25, TA Instruments, New Castle, DE, USA). About 10 mg of masterbatches were isothermal at −80 °C for 5 min. Then, the sample was heated from −80 °C to 30 °C at a heating rate of 6 °C/min, and the nitrogen purge was at a flow rate of 100 mL/min. The glass transition temperature (T_g_) is defined as the midpoint temperature of the glass transition approach. The normalized specific heat capacity step (∆C_pn_) and the mass fraction of immobilized polymer (χ_im_) are calculated as follows [50,51]:

∆C_pn_ = ∆C_p_/(1 − ω) (2)

χ_im_ = (∆C_p0_ − ∆C_pn_)/∆C_p0_ (3)

where ∆C_p_ is the change of specific heat capacity during glass transition, ω is the mass fraction of illite or K-illite, ∆C_p0_ indicates the specific heat capacity variation at T_g_ of unfilled SBR.

#### 2.5.6. Payne Effect

Payne effect was measured by Rubber Processing Analyzer (RPA2000, Alpha Technologies, Wilmington, DE, USA). The strain amplitude changed from 0.28% to 400%. The test frequency and temperature were 1 Hz and 60 °C, respectively. The compound sheet of 5 g was required for a test.

#### 2.5.7. Vulcanization Characteristics

The dynamic rheological properties during the curing process were determined by a moving die rheometer (M-3000A1, GOTECH, Zhuhai, China). The samples (5 g) were obtained from the compound sheet and the tests were performed at 160 °C with fixed frequency of 100 cpm (5/3 Hz) and oscillation amplitude of ± 0.5° according to ISO 3417-2008(E) standard.

#### 2.5.8. Morphology Observation and Elemental Analysis

The morphology of pristine illite and tensile fractured surface of vulcanizates were examined by a scanning electron microscope (Phenom ProX, Phenom World, Eindhoven, The Netherlands) at the operating voltage of 15 kV. Before observation, the illite and fractured surface were sputtered with a thin gold film. Elemental analysis of illite was characterized by energy-dispersive spectroscopy.

## 3. Results and Discussion

### 3.1. Characterization of Illite Particle Size and Specific Surface Area

**Figure 1:**
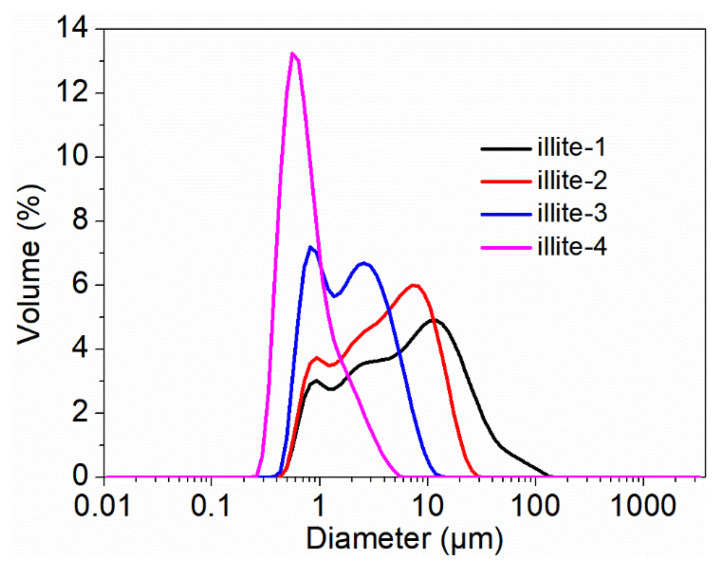
The particle size distribution of illite samples.

Particle size is one of the most vital factors affecting the reinforcement effect of fillers toward rubber. The particle size distribution of the used four kinds of illite samples are shown in Figure 1 with the corresponding size parameters and BET specific surface areas summarized in Table 2. It is clear that the illite-1 particles are the biggest among the used four illites with a size distribution ranging from 0.4 to 150 μm. The size distributions of illite-2 and illite-3 are approximately 0.4~30 μm and 0.4~13 μm, respectively. The illite-4 is the smallest with a size distribution ranging from 0.2 to 5 μm, indicating that the illite-3 particles were indeed broken down. It is the ultrasonic cavitation action between the interfaces of illite layers that distorts micro flakes slowly separated from the original aggregated sheets, leading to the decreasing of illite particle size [52]. The BET specific surface areas are accordingly increased with decreasing particle size.

### 3.2. Reinforcement of SBR by Illite or K-Illite with Different Particle Sizes

**Figure 2:**
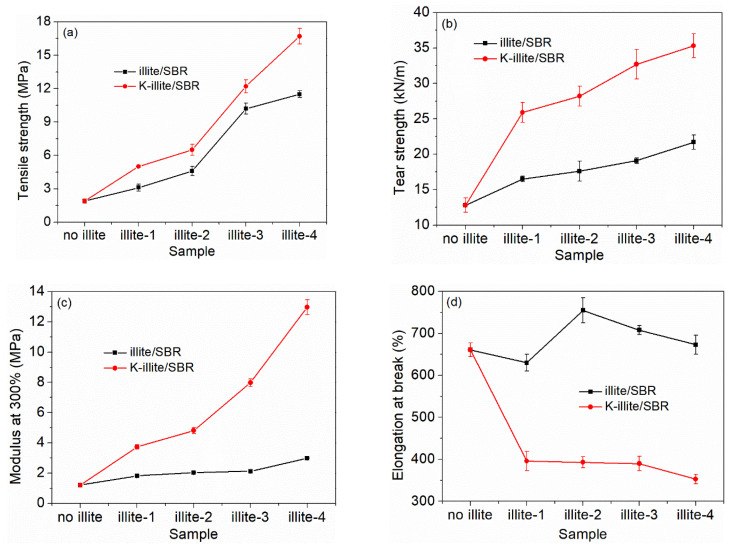
Mechanical properties of illite/SBR and K-illite/SBR vulcanizates filled with different particle sizes of filler. (**a**) tensile strength, (**b**) tear strength, (**c**) modulus at 300%, (**d**) elongation at break.

**Figure 3:**
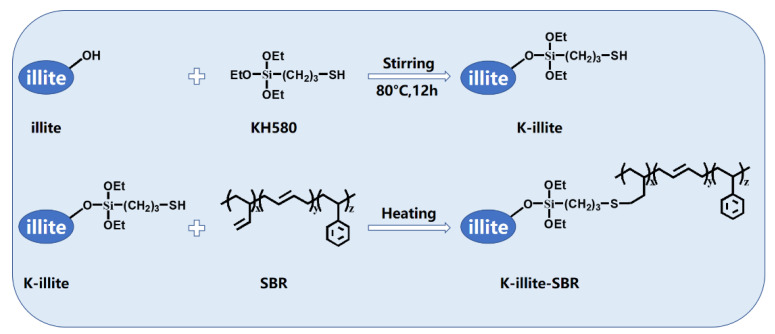
The proposed interaction between illite and SBR molecule in the incorporation of KH580.

Figure 2 shows the mechanical properties of illite/SBR and K-illite/SBR composites filled with different filler particles with detailed data summarized in Table 3. The typical stress–strain curves and related Young’s modulus and reinforcing factor are shown in Figure S2. From Figure 2a,b and Table 3, one can see that both the tensile and tear strengths of illite/SBR increase evidently with decreasing illite particle size. For example, the tensile and tear strengths increase from 1.9 MPa and 12.8 kN·m^−1^ for the unfilled SBR to 11.5 MPa and 21.7 kN·m^−1^ for illite-4/SBR vulcanizate, achieving an increment of 505% and 70%, respectively. Also, the modulus at 300% strain (Figure 2c) increases slightly with particle size. This illustrates the great effect of particle size on the properties of rubbers as frequently reported in the literature. For the elongation at break, it generally decreases as the modulus at 300% increases. The elongation at break of the illite/SBR composites is somewhat abnormal. As shown in Figure 2d, they are all close to the value of neat SBR with some undulation. The modification of illite by KH580 further improves the tensile and tear strengths, with an increase of 779% and 176% of the tensile (16.7 MPa) and tear (35.3 kN·m^−1^) strengths for K-illite-4/SBR being achieved with respect to the pure SBR. Even compared to the illite-4/SBR, the tensile and tear strengths of K-illite-4/SBR vulcanizate has increased by 45% and 63%, respectively. The improvement of the modulus at 300% strain of SBR by K-illite is more tremendous (Figure 2c). The modulus of illite-4/SBR is about two times the value of the SBR, while for K-illite-4/SBR, it is an order of magnitude higher than that of the SBR. The elongation at break of K-illite/SBR systems, however, drops more evidently. A better reinforcement efficiency of K-illite than illite toward SBR should be correlated to a stronger filler–rubber interaction caused by chemical bonding between K-illite and SBR chains as displayed in Figure 3, which has been confirmed by the results of FTIR measurement in Figure S3. Besides, the enhanced crosslinking density (as shown in Table 3) with the addition of K-illite in composites also accounts for the improved mechanical properties of K-illite/SBR vulcanizates.

The above results reveal clearly that, apart from the filler particle size, interfacial interaction between the filler and rubber is another important factor to determine the mechanical properties of rubber composites. It should be noticed here that the effect of interfacial interaction is more evident than the size decrease of the illite particle. Taken the tear strength and modulus as an example, the size decrease of illite results in an increment of the tear strength and modulus from 16.5 kN·m^−1^ and 1.82 MPa for illite-1/SBR to 21.7 kN·m^−1^ and 3.00 MPa for illite-4/SBR, namely an increase of 5.2 kN·m^−1^ and 1.18 MPa for illite-4, respectively. The modification of illite-4 with KH580 leads to a further increment of tear strength and modulus from 21.7 kN·m^−1^ and 3.00 MPa for illite-4/SBR to 35.3 kN·m^−1^ and 12.97 MPa for K-illite-4/SBR, respectively. Their increments are 13.6 kN·m^−^^1^ and 9.97 MPa, which are much larger than those caused by reduced particle size. Therefore, the synergistic effect of smaller size with larger specific surface area and stronger interfacial interaction achieved by KH580 modification makes the K-illite-4/SBR exhibits the best mechanical properties among all studied systems. By contrast, tensile strength, tear strength, and modulus at 300% of K-illite-4/SBR are superior to those of kaolinite (modified by bis-(γ-triethoxysilyl-propyl)-tetrasulfide coupling agent) filled SBR composite with the similar filler particle size, the same filler loading and the identical curing system (16.7 MPa, 35.3 kN/m, and 12.97 MPa vs. 11.48 MPa, 25.98 kN/m and 3.37 MPa, respectively) [21].

### 3.3. Dynamics of the Rubber Chain Segments during the Glass Transition

The stronger interfacial interaction will restrict the mobility of rubber chains or chain segments at illite surfaces. Thus, the filler–rubber interaction can be manifested by the rubber chain segments mobility at and near the illite particle surface. This has therefore been analyzed and presented below.

**Figure 4:**
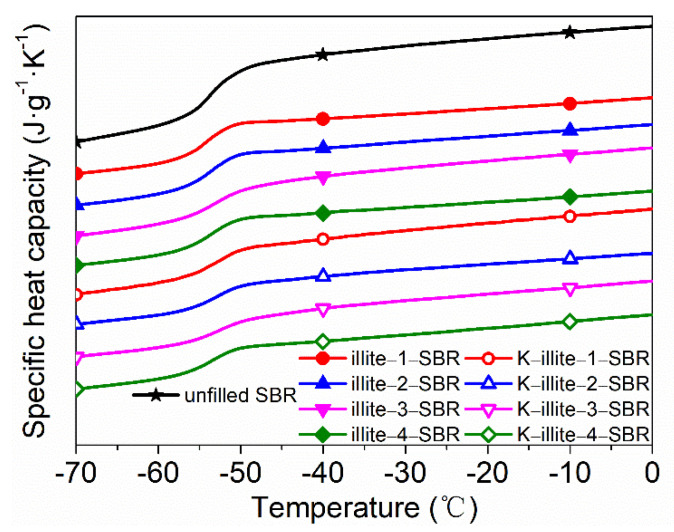
DSC thermograms showing the glass transition of SBR masterbatches.

Figure 4 shows the DSC thermograms of the SBR masterbatches. The related data during glass transition obtained from the DSC curves are listed in Table 4. It is seen that the glass transition temperatures (T_g_s) for all masterbatches are essentially the same within testing error. However, the change of specific heat capacity during glass transition (∆C_p_) is different for all samples. Generally, ∆C_p_ relies on the proportion of polymer participating in the glass transition and is relative to the degree of freedom of segmental motion. Furthermore, to eliminate the influence of polymer mass fraction, the normalized specific heat capacity step (∆C_pn_) is calculated.

As illustrated in Table 4, the values of ∆C_pn_ for illite-SBRs are all lower than those for unfilled SBR masterbatch, and meanwhile, decrease gradually with descending illite particle size. This originates from the restricted SBR chains or chain segments adsorbed onto the illite surface and the reduced mobility of the chain segments near interface due to the confinement effect. While the restricted chain segments dot not contribute to the glass transition at all, those with reduced mobility contribute less to the ∆C_pn_. As particles with smaller size provide more surface area for the SBR molecules, the ∆C_pn_ decreases consequently with decreasing particle size. In this sense, a further decrease of the ∆C_pn_ for K-illite-SBRs reflects an enhanced interaction between K-illite and SBR owing to the chemical bonding, which leads to a greater and stronger adsorption of the SBR chains. To quantitatively analyze the adsorbed chains, the mass fraction of immobilized polymer (χ_im_) has been calculated and summarized in Table 4. It has been utilized to evaluate the filler–rubber interaction of SBR composites.

It is clear that the χ_im_ increases gradually as the illite particle size decreases, demonstrating a greater adsorption of SBR chains on illite surfaces with increased areas. On the other hand, the larger χ_im_ values for K-illite-SBR masterbatches compared to the illite-SBR ones with identical size indicates a stronger filler–rubber interaction, since the bigger the χ_im_ is, the stronger the filler–rubber interaction. Taking all these into account, excellent mechanical properties of K-illite-4/SBR result clearly from a synergistic effect of particle size and filler–rubber interaction.

### 3.4. Payne Effect

**Figure 5:**
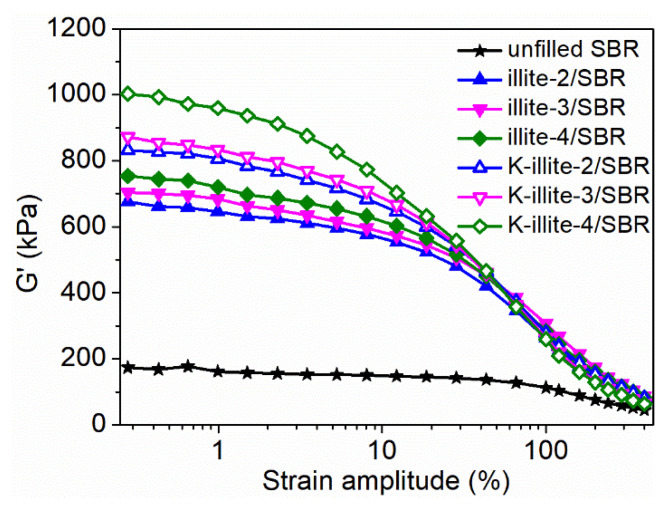
Strain amplitude-dependent nonlinear behavior of storage modulus (G′) for SBR compounds.

The Payne effect refers to the nonlinear behavior of storage modulus (G′) of rubber composites as the increasing of strain amplitude. It can be attributed to the breakdown of filler–filler and filler–rubber interaction. As shown in Figure 5, illite or K-illite filled SBR compounds all exhibit a typical Payne effect. It was reported that the hydroxyl groups of illite only exist at its edge surface, which accounts for only 10% of illite total surface [53], indicating that the agglomeration of illite in SBR matrix is not obvious because of the small number of its hydroxyl groups. Therefore, the illite–SBR interaction is mainly responsible for the Payne effect of (K-)illite/SBR compounds. The higher G′ at low strain amplitudes for K-illite/SBR compounds reflects that they have a stronger illite–rubber interaction than illite/SBR, which is due to the chemical bonding between K-illite and SBR chains. Meanwhile, the enhanced interface bonding leads to more entanglements with rubber chains. The debonding and disentanglement of SBR chains at and near the compound interface under high strain amplitudes result in the nonlinear viscoelasticity of K-illite/SBR. As the illite particle size decreases, the values of G′ at low strain amplitudes for both illite/SBR and K-illite/SBR are increased. The increasing specific surface area causes the possible enhancement of filler network, and more importantly, more adsorption of rubber chains onto illite surface, contributing to the stronger illite–rubber interaction. These both bring about a larger G′ at low strain amplitudes.

### 3.5. Morphology of Illite/SBR and K-Illite/SBR Vulcanizates

**Figure 6:**
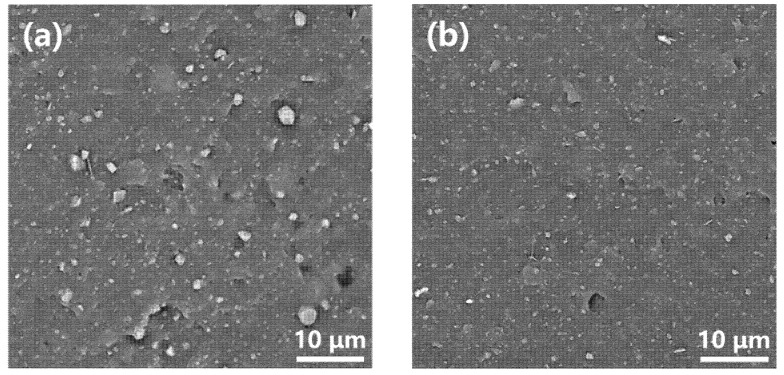
Observation of tensile fractured surface of vulcanizates by SEM (**a**) illite-4/SBR, (**b**) K-illite-4/SBR.

It is widely accepted that a stronger filler–rubber interaction favors the dispersion of the fillers in the rubber matrix. Therefore, a better dispersion of K-illite in SBR matrix than the illite is highly expected. To check this, the tensile fractured surfaces of illite/SBR and K-illite/SBR vulcanizates were visually examined by scanning electron microscopy (SEM) to illustrate the stronger interaction between K-illite and SBR. The representative micrographs of illite-4/SBR and K-illite-4/SBR are presented in Figure 6a,b, respectively. It can be seen that abundant illite particles with varied size are exposed on the fractured surface. The average size of the illite observed on the illite-4/SBR surface (Figure 6a) is relatively larger than on the K-illite-4/SBR surface (Figure 6b), indicating really a better dispersion of K-illite. Moreover, while the K-illites are well connected with the SBR matrix, there exist obvious gaps between the illite and SBR matrix. This provides another indicator for an enhanced interaction between K-illite and SBR matrix.

### 3.6. Dynamic Rheological Properties during Curing Process

**Figure 7:**
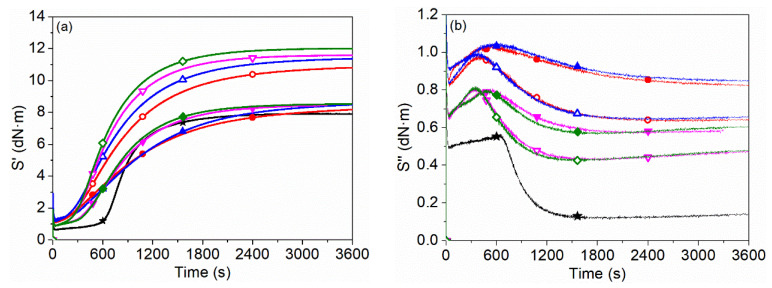
Curing curves of (**a**) elastic torque (S′) and (**b**) viscous torque (S″) for (K-)illite filled SBR composites. The solid represents illite/SBR, while the hollow represents K-illite/SBR. The red, blue, magenta, and olive lines indicate (K-)illite-1, (K-)illite-2, (K-)illite-3 and (K-)illite-4 filled SBR composites, respectively. The black line indicates unfilled SBR composite.

Rheological behaviors of illite/SBR and K-illite/SBR composites during the curing process were studied. The elastic torque (S′) and viscous torque (S″) as presented in Figure 7a,b, respectively, are closely related to the particle size and filler–rubber interaction. The curing characteristics including the maximum torque (M_H_), the minimum torque (M_L_), the scorch time (t_10_, the time to achieve 10% of full torque development) and the optimum curing time (t_90_, the time to achieve 90% of full torque development) are summarized in Table 5. The t_10_ and t_90_ can be used to estimate the safety and efficiency of rubber processing, respectively [21]. It can be observed that the t_10_ of all illite/SBR and K-illite/SBR composites is shorter than that of the unfilled SBR, while their t_90_s get longer compared with unfilled SBR. The decrease of t_10_ is probably due to the moisture-treated hydroxyl groups on illite surface and/or the hydrolysis of nitrogen–sulfur bonds in the accelerator NS, which restrains its scorch-delay action and produces a faster onset of vulcanization [54,55]. The lengthening of t_90_ is attributed to the adsorption effect of illite on accelerator, resulting in the delay of curing. Fortunately, the modification of illite by KH580 reduces the adsorption of accelerator onto the illite surface, causing a shorter t_90_ of K-illite/SBR than that of illite/SBR composites. However, only the SBR composite filled with K-illite-4 (t_90_ = 23.27 min), which has the smallest particle size among the four illite samples, that reaches a slightly higher vulcanization efficiency than that of unfilled SBR (t_90_ = 23.73 min). Even so, the decrement of particle size for illite is beneficial to the promotion of curing process no matter the incorporation of KH580 or not, according to the decreasing tendency of t_90_ with the reduced particle size, especially for SBR composites filled with illite-3 and illite-4, a significant gradient of descent can be found. The acceleration of curing due to the filler particle size reduction is ascribed to the better interaction between the smaller filler and rubber matrix [56]. The t_10_, however, has no obvious variations as the reduction of illite particle size. It is inferred that both the reduction of particle size and the modification of illite by KH580 lead to the improved vulcanization efficiency, which becomes the reason for the increase of crosslinking density as shown in Table 3.

The M_H_ and M_L_, which display the stock modulus of rubber composites and the viscosity of rubber [21,57], respectively, have an increment by various degrees for illite/SBR and K-illite/SBR composites compared with those of unfilled SBR, attributing to the addition of illite particles. M_H_-M_L_, which stands for the rigidity of rubber composites dating from the filler dosage, crosslinking density and filler–rubber interaction [58] increases gradually with the reduction of illite particle size. As the dosage of illite is constant, M_H_-M_L_ is mainly determined by crosslinking density and filler–rubber interaction. So, except for the increased crosslinking density, it reflects that the interfacial interaction between illite and SBR molecules also increases with the decrease of illite particle size, because the more specific surface area, the more rubber molecules will be adsorbed on the illite surface, causing the more restriction of mobility for SBR chains. In addition, for the SBR composites filled by (K-)illite with the same particle size, M_H_-M_L_ exhibits a rising tendency after the modification of illite by KH580, which is caused by the synergistic effect of the enhanced filler–rubber interaction and the increased crosslinking density. The formation of chemical bonds between illite and SBR chains in the presence of KH580 is the main reason for the growing crosslinking density of K-illite/SBR compared with illite/SBR vulcanizates. As for illite/SBR composites, the slightly lower M_H_-M_L_ of illite-1/SBR than unfilled SBR may be due to the existence of illite particles larger than 40 μm, which have little reinforcement on rubber but instead introduce flaws in rubber matrix and cause the breakage of rubber [20]. These may also explain the abnormal elongation at break for illite-1/SBR vulcanizate in Figure 2d. In contrast, larger M_H_-M_L_ for other illite/SBR composites than unfilled SBR is always observed, though with smaller crosslinking density compared to the unfilled SBR, which further highlights the importance of filler–rubber interaction.

The S″ curves displayed in Figure 7b have a dramatic decrease in about 30 s as a result of the improved mobility of SBR chains with the sharp rise in temperature. Then they increase until a peak appears as time goes on, followed by a decline and finally flat tendency. In contrast to the S′ curves, it can be seen that the peak time in S″ curve is almost identical to the scorch time in S′ curve, which is the line of demarcation between the induction period and the curing period in curing process. Therefore, the peak time of filled SBR composites is shorter than that of unfilled SBR due to their smaller t_10_. Moreover, the modification of illite by KH580 makes the peak time of K-illite/SBR composites shorter than that of illite/SBR, attributed to the increasing curing efficiency with shorter t_10_ in the existence of KH580.

## 4. Conclusions

In summary, the reinforcement effect of naturally-occurring and low-carbon illite with different particle sizes and their KH580 modified counterpart K-illite as fillers for SBR were studied. The results are as follows. (1) The mechanical properties of SBR can be improved both by raw illite and modified K-illite. The better reinforcement of K-illite than illite toward SBR is observed and confirmed to be attributed to the enhanced interaction of K-illite with SBR chains through chemical bonding, which also promotes the dispersion of K-illite in the SBR matrix. (2) The interfacial interaction and mechanical properties increase with reduction of particle size owing to increased specific surface area. (3) The K-illite-4/SBR composite has the best performances, whose tensile strength, tear strength and modulus at 300% are 16.7 MPa, 35.3 kN/m and 12.97 MPa, respectively, which are superior to those of kaolinite (modified by bis-(γ-triethoxysilyl-propyl)-tetrasulfide coupling agent) filled SBR composite under similar conditions. Further efforts are required for illite to endow the rubber composite with better properties than carbon black. Finding alternatives to non-renewable carbon black filler is of great significance for achieving sustainable development in the rubber industry.

## Figures and Tables

**Table 1:** The basic formulation (per hundred parts of rubber, phr).

Sample	SBR	ZnO	SA	NS	S	Illite	KH580
unfilled SBR	100	3	1	1	1.75	0	0
illite-x/SBR	100	3	1	1	1.75	40	0
K-illite-x/SBR	100	3	1	1	1.75	40	4

x = 1, 2, 3, 4, representing the used illite.

**Table 2:** Parameters of four different illite particles.

Sample	D_10_/μm	D_50_/μm	D_90_/μm	S_BET_/m^2^·g^−1^
illite-1	1.02 ± 0.00	6.24 ± 0.02	25.80 ± 0.20	13 ± 0.2
illite-2	0.94 ± 0.00	4.10 ± 0.01	12.30 ± 0.00	15 ± 0.3
illite-3	0.71 ± 0.01	1.88 ± 0.01	5.14 ± 0.02	21 ± 0.1
illite-4	0.42 ± 0.00	0.70 ± 0.00	1.82 ± 0.01	50 ± 0.3

**Table 3:** Mechanical properties and crosslinking density of different vulcanizates.

Sample	Tensile Strength /MPa	Tear Strength /kN·m^−1^	Modulus at 300% /MPa	Elongation at Break /%	XLD ^1^ /10^−5^mol/cm^3^
SBR	1.9 ± 0.2	12.8 ± 1.0	1.21 ± 0.03	661 ± 16	6.37 ± 0.12
illite-1/SBR	3.1 ± 0.3	16.5 ± 0.4	1.82 ± 0.02	630 ± 20	2.54 ± 0.11
illite-2/SBR	4.6 ± 0.4	17.6 ± 1.4	2.04 ± 0.02	755 ± 30	2.68 ± 0.15
illite-3/SBR	10.2 ± 0.5	19.1 ± 0.4	2.12 ± 0.04	708 ± 11	4.32 ± 0.13
illite-4/SBR	11.5 ± 0.3	21.7 ± 1.0	3.00 ± 0.05	673 ± 23	4.69 ± 0.09
K-illite-1/SBR	5.0 ± 0.1	25.9 ± 1.4	3.73 ± 0.15	396 ± 23	7.06 ± 0.24
K-illite-2/SBR	6.5 ± 0.5	28.2 ± 1.4	4.81 ± 0.19	393 ± 13	8.41 ± 0.25
K-illite-3/SBR	12.2 ± 0.6	32.7 ± 2.1	7.98 ± 0.25	390 ± 17	10.07 ± 0.38
K-illite-4/SBR	16.7 ± 0.7	35.3 ± 1.7	12.97 ± 0.49	353 ± 11	11.88 ± 0.27

^1^ XLD represents the crosslinking density of vulcanizates.

**Table 4:** Related data during glass transition obtained from DSC curves.

Sample	T_g_/°C	∆C_pn_/ J·g^−1^·K^−1^	χ_im_
unfilled SBR	−53	0.343	—
illite-1-SBR	−54	0.306	0.109
illite-2-SBR	−54	0.296	0.138
illite-3-SBR	−54	0.289	0.158
illite-4-SBR	−55	0.281	0.182
K-illite-1-SBR	−53	0.255	0.258
K-illite-2-SBR	−53	0.243	0.291
K-illite-3-SBR	−53	0.238	0.307
K-illite-4-SBR	−54	0.224	0.346

**Table 5:** Cure characteristics of illite/SBR and K-illite/SBR composites with different illite particle sizes.

Sample	M_H_/dN·m	M_L_/dN·m	M_H_-M_L_ /dN·m	t_10_/min	t_90_/min
unfilled SBR	7.77	0.63	7.14	10.50	23.73
illite-1/SBR	8.13	1.13	7.00	5.88	37.85
illite-2/SBR	8.43	1.27	7.16	5.98	37.95
illite-3/SBR	8.29	0.89	7.40	6.30	27.90
illite-4/SBR	8.37	0.87	7.50	6.57	26.55
K-illite-1/SBR	10.67	1.09	9.58	5.12	31.78
K-illite-2/SBR	11.17	1.16	10.01	4.75	28.88
K-illite-3/SBR	11.36	0.88	10.48	5.07	24.20
K-illite-4/SBR	11.77	0.89	10.88	5.08	23.27

## Data Availability

Not applicable.

## Supplementary Materials

The following supporting information can be downloaded at: https://www.mdpi.com/article/10.3390/ma15103459/s1, Figure S1: (a) The macroscopic feature (illite powder), (b) microcosmic feature (scanning electron micrograph) and (c) elemental analysis of illite-3; Figure S2: (a) Stress–strain curves of vulcanizates during tensile process, (b) Young’s modulus obtained from the stress–strain curves and (c) reinforcing factor calculated by the ratio of modulus at 300% to modulus at 100%. 0, 1, 2, 3, 4 represent unfilled SBR, (K-)illite-1/SBR, (K-)illite-2/SBR, (K-)illite-3/SBR, (K-)illite-4/SBR vulcanizates, respectively; Figure S3: FTIR spectra of raw illite-4 (black line) and K-illite-4 (red line). References [59,60] are cited in the supplementary materials.
