# Supplementary material for: Effect of 3-Mercaptopropyltriethoxysilane Modified Illite on the Reinforcement of SBR
Source: Materials (Basel). 2022 May 11;15(10):3459. doi: 10.3390/ma15103459 (PMC9143291; doi:10.3390/ma15103459)
Supplement: Supplementary file 1 [file materials-15-03459-s001.zip › materials-1694794-supplementary.pdf]

Supplementary materials

Effect of 3-mercaptopropyltriethoxysilane (KH580) modified illite on the reinforcement of SBR

Zhepeng Wang [1], Hao Zhang [1,*], Qiang Liu [1], Shaojuan Wang [1] and Shouke Yan [1,2,*]

1 Key Laboratory of Rubber-Plastics, Ministry of Education, Qingdao University of Science & Technology, Qingdao 266042, China; wzhepeng@163.com (Z.W.); zhanghao@qust.edu.cn (H.Z.); liuqiang@qust.edu.cn (Q.L.); wangshaojuan1982@163.com (S.W.); skyan@qust.edu.cn (S.Y.)

2 State Key Laboratory of Chemical Resource Engineering, Beijing University of Chemical Technology, Beijing 100029, China; skyan@mail.buct.edu.cn (S.Y.)

* Correspondence: skyan@mail.buct.edu.cn or skyan@qust.edu.cn (S.Y.); zhanghao@qust.edu.cn (H.Z.)

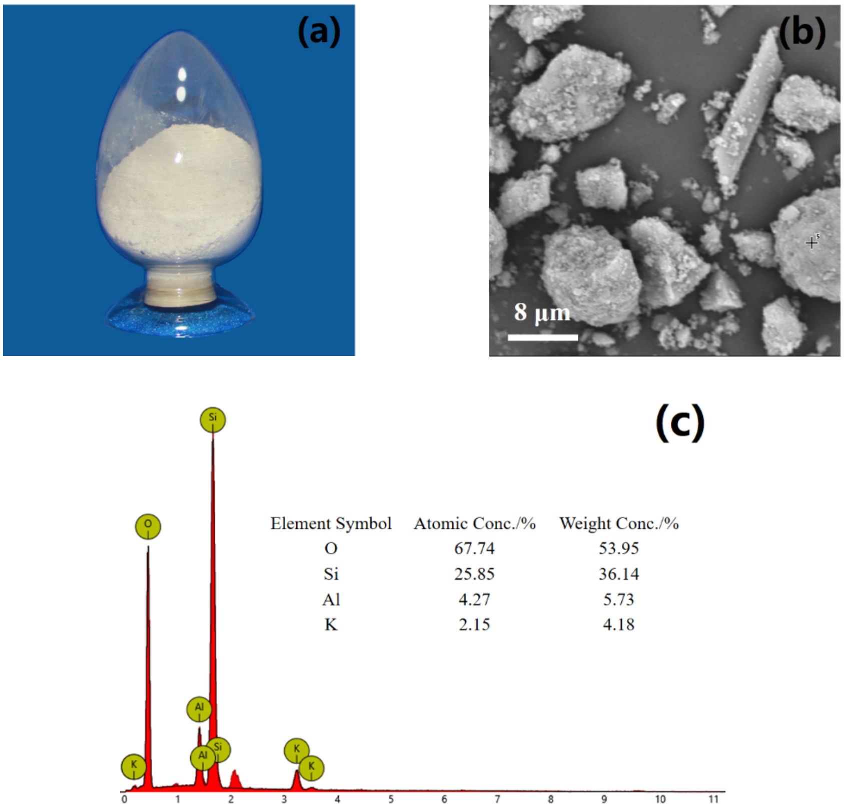

Figure S1. (a) The macroscopic feature (illite powder), (b) microcosmic feature (scanning electron micrograph) and (c) elemental analysis of illite-3.

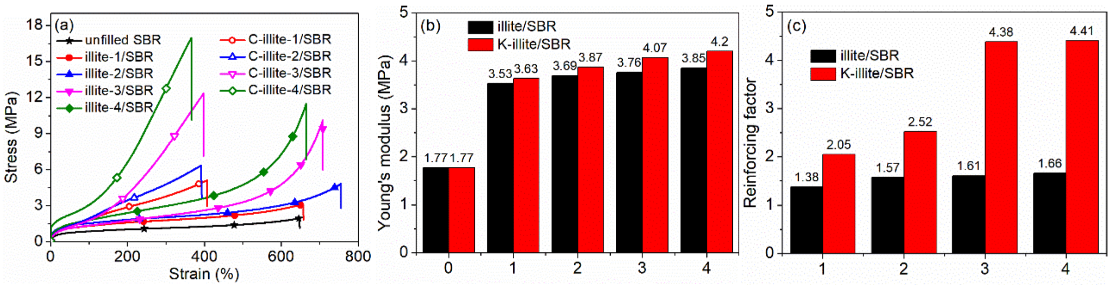

Figure S2. (a) Stress–strain curves of vulcanizates during tensile process, (b) Young's modulus obtained from the stress–strain curves and (c) reinforcing factor calculated by the ratio of modulus at 300% to modulus at 100%. 0, 1, 2, 3, 4 represent unfilled SBR, (K-)illite-1/SBR, (K-)illite-2/SBR, (K-)illite-3/SBR, (K-)illite-4/SBR vulcanizates, respectively.

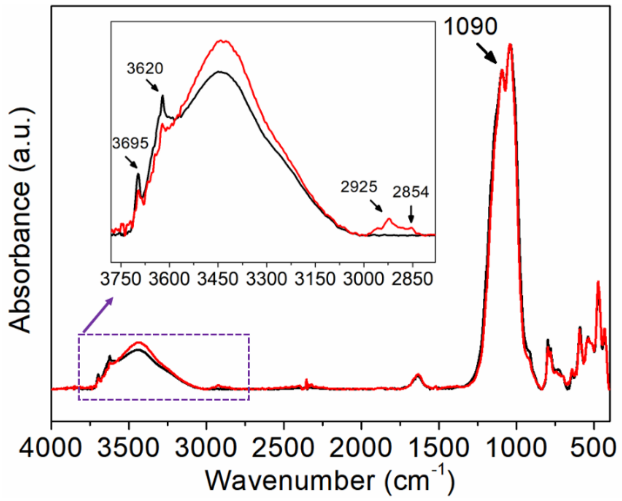

Figure S3. FTIR spectra of raw illite-4 (black line) and K-illite-4 (red line).

Figure S3 shows the FTIR spectra of raw illite-4 and KH580 modified illite-4 (K-illite-4). The bands were normalized with respect to the band at 1090 cm^{-1} attributing to the free Si–O vibration [59]. It can be seen from the inset that new bands at 2925 cm^{-1} and 2854 cm^{-1} appear on the spectrum of K-illite in contrast to that of illite. They are assigned to the antisymmetric and symmetric stretching vibration of C–H in KH580, respectively, indicating the successfully grafting of KH580 on illite. Besides, the intensities of the characteristic bands of illite at 3695 cm^{-1} and 3620 cm^{-1} decrease due to the KH580 modification. They correspond to the stretching vibration of hydroxyl groups at the edge surface and inner hydroxyl groups locating between the tetrahedron and octahedron layers of illite [60], respectively, which demonstrates that grafting reaction occurs between the KH580 and the hydroxyl groups of illite. These confirm that KH580 has effectively bonded to the illite surface.
